# Mapping the landscape: a bibliometric analysis of resting-state fMRI research on schizophrenia over the past 25 years

**DOI:** 10.1038/s41537-024-00456-2

**Published:** 2024-03-15

**Authors:** Linhan Fu, Remilai Aximu, Guoshu Zhao, Yayuan Chen, Zuhao Sun, Hui Xue, Shaoying Wang, Nannan Zhang, Zhihui Zhang, Minghuan Lei, Ying Zhai, Jinglei Xu, Jie Sun, Juanwei Ma, Feng Liu

**Affiliations:** 1 Department of Radiology and Tianjin Key Laboratory of Functional Imaging, Tianjin Medical University General Hospital, Tianjin, 300052 China; 2 School of Medical Imaging, Tianjin Medical University, Tianjin, 300070 China; 3 School of Medicine, Nankai University, Tianjin, 300071 China

**Keywords:** Schizophrenia, Biomarkers

## Abstract

Schizophrenia, a multifaceted mental disorder characterized by disturbances in thought, perception, and emotion, has been extensively investigated through resting-state fMRI, uncovering changes in spontaneous brain activity among those affected. However, a bibliometric examination regarding publication trends in resting-state fMRI studies related to schizophrenia is lacking. This study obtained relevant publications from the Web of Science Core Collection spanning the period from 1998 to 2022. Data extracted from these publications included information on countries/regions, institutions, authors, journals, and keywords. The collected data underwent analysis and visualization using VOSviewer software. The primary analyses included examination of international and institutional collaborations, authorship patterns, co-citation analyses of authors and journals, as well as exploration of keyword co-occurrence and temporal trend networks. A total of 859 publications were retrieved, indicating an overall growth trend from 1998 to 2022. China and the United States emerged as the leading contributors in both publication outputs and citations, with Central South University and the University of New Mexico being identified as the most productive institutions. Vince D. Calhoun had the highest number of publications and citation counts, while Karl J. Friston was recognized as the most influential author based on co-citations. Key journals such as Neuroimage, Schizophrenia Research, Schizophrenia Bulletin, and Biological Psychiatry played pivotal roles in advancing this field. Recent popular keywords included support vector machine, antipsychotic medication, transcranial magnetic stimulation, and related terms. This study systematically synthesizes the historical development, current status, and future trends in resting-state fMRI research in schizophrenia, offering valuable insights for future research directions.

## Introduction

Schizophrenia is a complex mental disorder characterized by abnormalities in cognition, perception, and emotion^1,2^. Patients may experience hallucinations, delusions, emotional detachment, reduced motivation, social withdrawal, and cognitive decline^3,4^. Moreover, these impairments frequently result in adverse clinical consequences, including unemployment, inability to maintain independent living, and long-term disability. Despite extensive endeavors to elucidate its etiology, symptomatology, and therapeutic approaches, the biological foundation and neural mechanisms of schizophrenia remain largely elusive.

Over the past decade, functional neuroimaging technology has played an increasingly crucial role in examining the neurobiological foundations of schizophrenia^5,6^. Among these methodologies, resting-state fMRI emerges as highly promising, as it avoids potential biases linked to specific tasks, demands minimal participant effort, and is relatively easy to implement in clinical contexts^7,8^. Recent years have witnessed a surge in resting-state fMRI studies investigating neural abnormalities in schizophrenia. Employing various metrics such as regional homogeneity^9,10^, amplitude of low-frequency fluctuation^11,12^, functional connectivity^13,14^, and functional network analysis^15,16^, researchers have unveiled widespread resting-state functional alterations in individuals with schizophrenia. For example, individuals with schizophrenia exhibit decreased spontaneous activity in the bilateral postcentral gyri and paracentral lobule, which has been linked to cognitive impairment in this population^17^. Additionally, prior research has identified significant functional disconnections within default mode network in patients with schizophrenia, negatively correlating with positive and mood symptoms^18^. Moreover, in schizophrenia, functional network analysis has revealed disrupted small-world topological characteristics, with the altered topological measurements correlating with the duration of illness^19^. Despite these findings, the field of resting-state fMRI research in schizophrenia remains far from being fully explored. In particular, there has been little systematic examination of research hotspots and emerging trends of resting-state fMRI for schizophrenia.

Bibliometrics, as a quantitative method, plays a crucial role in evaluating the evolution of a field, identifying influential works, and revealing emerging trends, thereby enabling a comprehensive understanding of the dynamics within a research domain^20–22^. The analysis of publication patterns, citation networks, and author collaborations serves to identify influential studies and authors, guide future research and collaboration efforts, and promote interdisciplinary approaches for addressing complex issues like schizophrenia. Ultimately, this contributes to advancements in understanding, diagnosing, and treating the disorder, driving progress in the field^23^. However, to date, no bibliometric analysis has been conducted on the publication patterns of resting-state fMRI studies associated with schizophrenia.

In this study, bibliometric approaches were employed to analyze academic publications spanning the past 25 years, focusing on resting-state fMRI studies related to schizophrenia. The purpose was mainly twofold: firstly, we aimed to leverage bibliometric techniques to conduct a comprehensive literature review of resting-state fMRI research on schizophrenia, involving the identification of leading countries/regions and institutions, influential authors, and key research themes that have shaped the field’s trajectory. Secondly, we sought to address critical knowledge gaps and spotlight emerging research trends within this domain, aiming to facilitate a deeper understanding of the neural mechanisms underpinning schizophrenia through a holistic overview of the research landscape in resting-state fMRI studies.

## Materials and methods

### Data acquisition

**Fig. 1:**
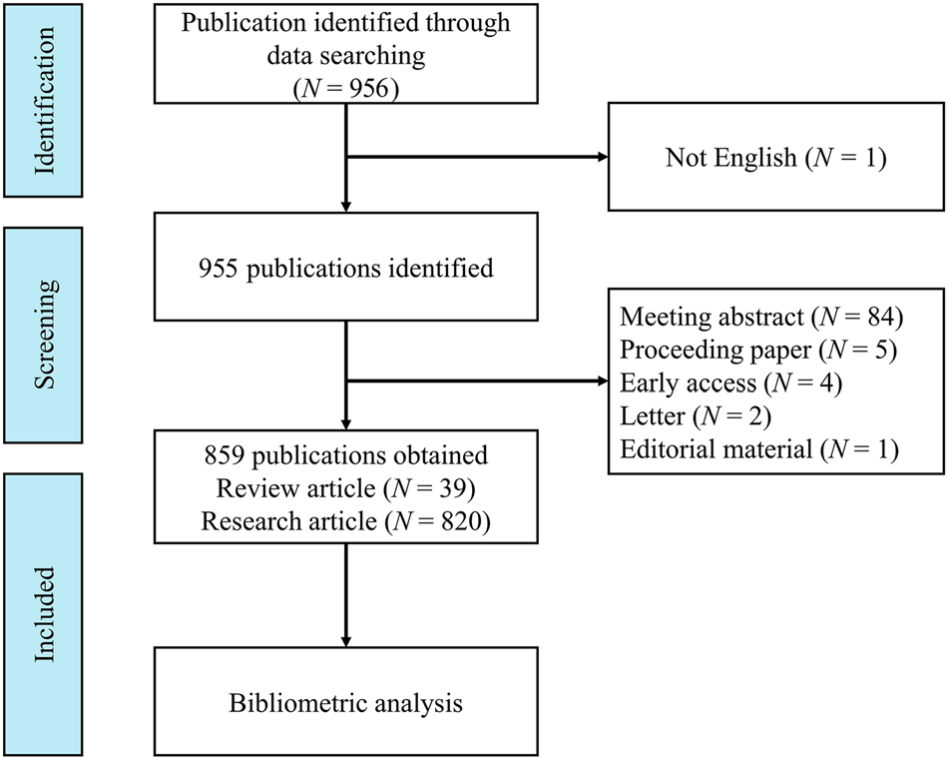
The flowchart of literature search and selection in the bibliometric analysis of resting-state fMRI research on schizophrenia.

The study was carried out by utilizing the Web of Science Core Collection, recognized as a leading database for bibliometric analyses^24^, which encompasses a substantial amount of high-quality scientific literature. The search strategy included the following terms: TS = (“resting-state” or “rest” or “resting”) AND TS = (“functional magnetic resonance imaging” or “functional MRI” or “fMRI”) AND TI = (“Schizophrenia” or “Schizophreniform” or “Schizoaffective” or “Schizophrenic Disorder” or “Schizophrenic”). The study period was restricted to 1998–2022, and the search was limited to English-language articles and review articles. Various document types, including meeting abstracts, proceedings papers, early access materials, letters, and editorial content, were excluded. Articles lacking full texts and duplicate publications were also excluded. The full record and cited references were exported as plain text files, with the comprehensive search process outlined in Fig. 1.

### Data analysis

Each article underwent a thorough review process, during which details such as the author, country, institution, publication year, title, journal, reference, and keyword were carefully recorded. Data on the number of publications and citations, as well as journal impact factors, were obtained from Web of Science. Additionally, co-citation count, defined as the frequency with which two published documents are cited by the same third document^25^, was employed in the analysis. Moreover, co-authorship analysis was conducted to evaluate scientific collaboration among countries/regions, institutions, and authors by examining the frequency of co-authorship, and co-citation analysis was performed to identify influential authors based on their proximity. Furthermore, keyword co-occurrence, measuring the frequency of keywords appearing together, served as a valuable tool for detecting research fronts.

The VOSviewer software^26^, developed by Leiden University in the Netherlands, was utilized to construct and visualize co-authorship networks of countries/regions, institutions, and authors, co-citation networks of authors and journals, as well as keyword co-occurrence networks. The VOS technique employed a measure of association known as the VOS mapping to assign keywords to clusters based on their frequency of occurrence within those clusters^27^. Each node in the network visualization represented an item, such as a country/region, institution, author, journal, or keyword. The color of nodes denoted different clusters, while the size of the node reflected the number of occurrences or citations, with larger nodes indicating more citations or occurrences. Lines between nodes illustrated the links between terms, with the thickness of the line indicating the strength of the link^28^. In this study, total link strength (TLS) was utilized to quantify the strength of connections, reflecting the overall strength of links with other items.

## Results

### Annual publications and citations

**Fig. 2:**
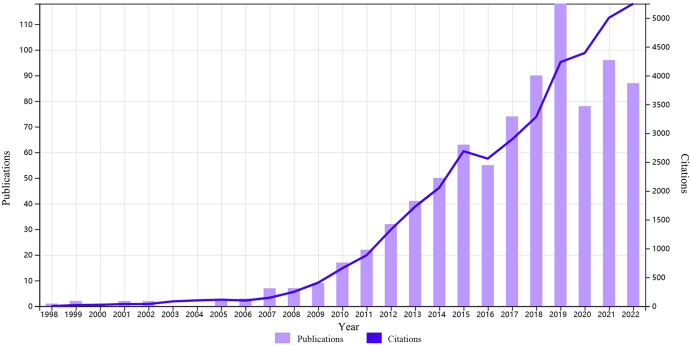
Trends in annual publications and citations in resting-state fMRI research related to schizophrenia (1998–2022).

The search collected publication and citation data of schizophrenia-related literature based on resting-state fMRI spanning from 1998 to 2022, resulting in the inclusion of a total of 859 publications after excluding irrelevant articles. For the detailed information of all included studies, please see the Supplementary file. The overall trend in publication output is shown in Fig. 2, depicting a gradual increase from 1 article in 1998 to 87 in 2022. Simultaneously, the number of citations for these publications increased from 0 to 5252 by the year 2022. Prior to 2010, the annual publication output remained below ten articles, accompanied by relatively low citation counts. However, after 2010, research in the field experienced a rapid expansion, with publication output surging more than 5-fold from 17 articles in 2010 to 87 articles in 2022, and citations increasing nearly 8-fold from 657 times in 2010 to 5252 times in 2022. Although there was a decrease in the number of publications in 2020, it rebounded in 2021 and 2022.

### Analysis of country/region

In total, the literature analyzed originated from 51 different countries or regions. The top 15 countries/regions were ranked by the number of published papers, as shown in Table S1, with China leading with 399 papers, closely followed by the United States with 374, and England in third place with 80 papers. The fourth to tenth positions were occupied by Canada, Germany, Italy, the Netherlands, Spain, Australia, and South Korea, respectively. In terms of citation counts, the United States ranked first with 23,821 citations, followed by China with 12,235 citations in second place, and England in third place with 7916 citations.

**Fig. 3:**
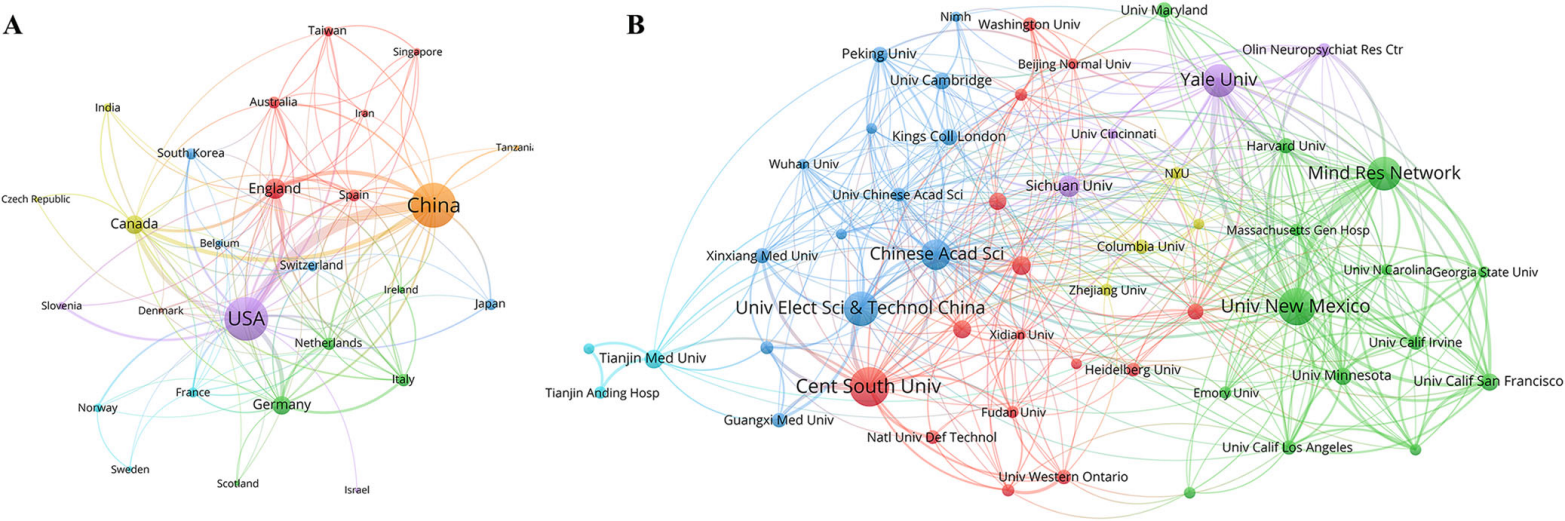
Country/region and institution collaboration maps (co-authorship maps) in resting-state fMRI research related to schizophrenia. **A** Country/region collaboration map. **B** Institution collaboration map. Each node represents one country/region or institution, and the size of the node is proportional to the number of publications from the country/region or institution. The lines connecting two nodes indicates they have at least one publication in common, with thicker lines indicating a greater number of co-authors between two countries/regions or institutions.

Additionally, we established a collaboration network among countries/regions (co-authorship network) by selecting the 27 countries/regions that had published at least five papers. The analysis results of the cooperation network of countries/regions indicated that collaboration between different countries/regions was highly active in this field (Fig. 3A). The United States had the strongest international collaboration network (with a TLS of 306), followed by China (244), England (124), and Canada (101). The link between China and the United States was the strongest, indicating the most significant collaboration between these two countries. Furthermore, both China and the United States maintained active collaborations with several other countries, including England and Canada.

### Analysis of institution

According to Table S2, Central South University emerged as the top publisher of articles on resting-state fMRI research in schizophrenia, with 91 documents, followed by the University of New Mexico with 85 documents, and the University of Electronic Science and Technology of China with 75 documents. In terms of citations, Yale University secured the top spot with 7710 citations, followed closely by the University of New Mexico with 7307 citations and the Mind Research Network with 4468 citations. Other significant contributors included Central South University (4373 citations), Chinese Academy of Sciences (4299 citations), University of Cambridge (3696 citations), and King’s College London (2172 citations). Collaboration between institutions is displayed in Fig. 3B, with the University of New Mexico, the Mind Research Network, and Yale University showing the highest TLS.

### Analysis of author and co-cited authors

**Fig. 4:**
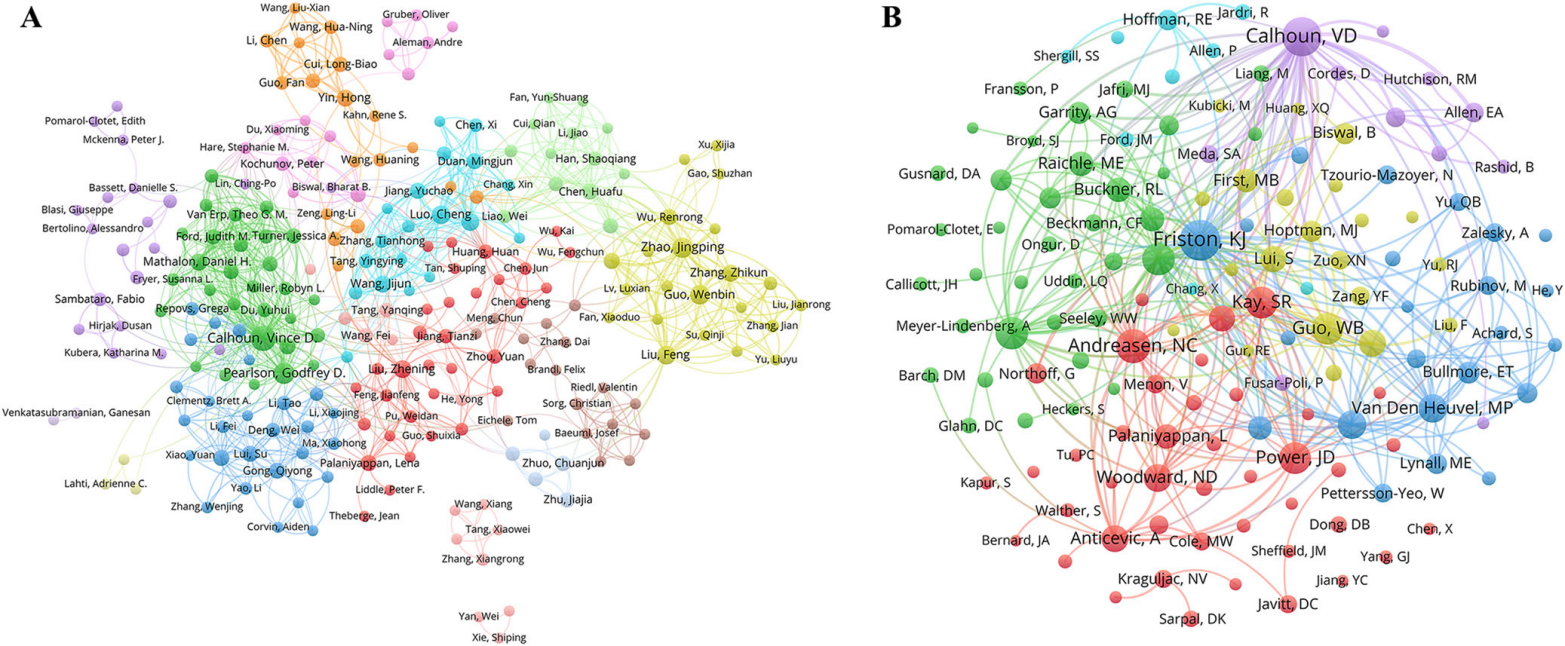
The network map of authors engaged in resting-state fMRI research related to schizophrenia. **A** Co-authorship map. Each node represents one author, with larger nodes indicating more numbers of co-authors the author has. The lines connecting two nodes indicates they have at least five publications in common, with thicker lines indicating a greater number of co-authors between two authors. **B** Co-citation map. Each node represents one author, with larger nodes indicating more citations. Lines between nodes demonstrate the number of their published documents cited by the same third document, with thicker line indicating a higher number of co-citations between them.

The top 15 authors ranked by the number of publications are listed in Table S3. Vince D. Calhoun had the most publications with 80 followed by Godfrey D. Pearson (44), Jingping Zhao (38), Wenbin Guo (35), and Cheng Luo (29). Co-authorship networks offered a clear view of collaboration patterns among authors, utilizing the frequency of co-authorship. With a threshold of at least five publications per author, the co-authorship network included 257 authors out of 3914 authors (Fig. 4A). In the co-citation map (Fig. 4B), the number of co-citations was derived from reference citations in papers, serving as an indicator of an author’s academic influence and recognition by peers. The ranking of authors in the co-citation map is presented in Table S4, determined by both their co-citation numbers and TLS. Among the researchers analyzed, Karl J. Friston led with the highest co-citation count at 497 and TLS of 11,246. Following closely, Vince D. Calhoun had 451 co-citations and a TLS of 11,242. In addition, Nancy C. Andreasen, Susan Whitfield-Gabrieli, and Michael D. Fox were influential with 362, 326, and 321 co-citations, accompanied by TLS of 7418, 7564, and 8314, respectively.

### Analysis of journal and co-cited journals

**Fig. 5:**
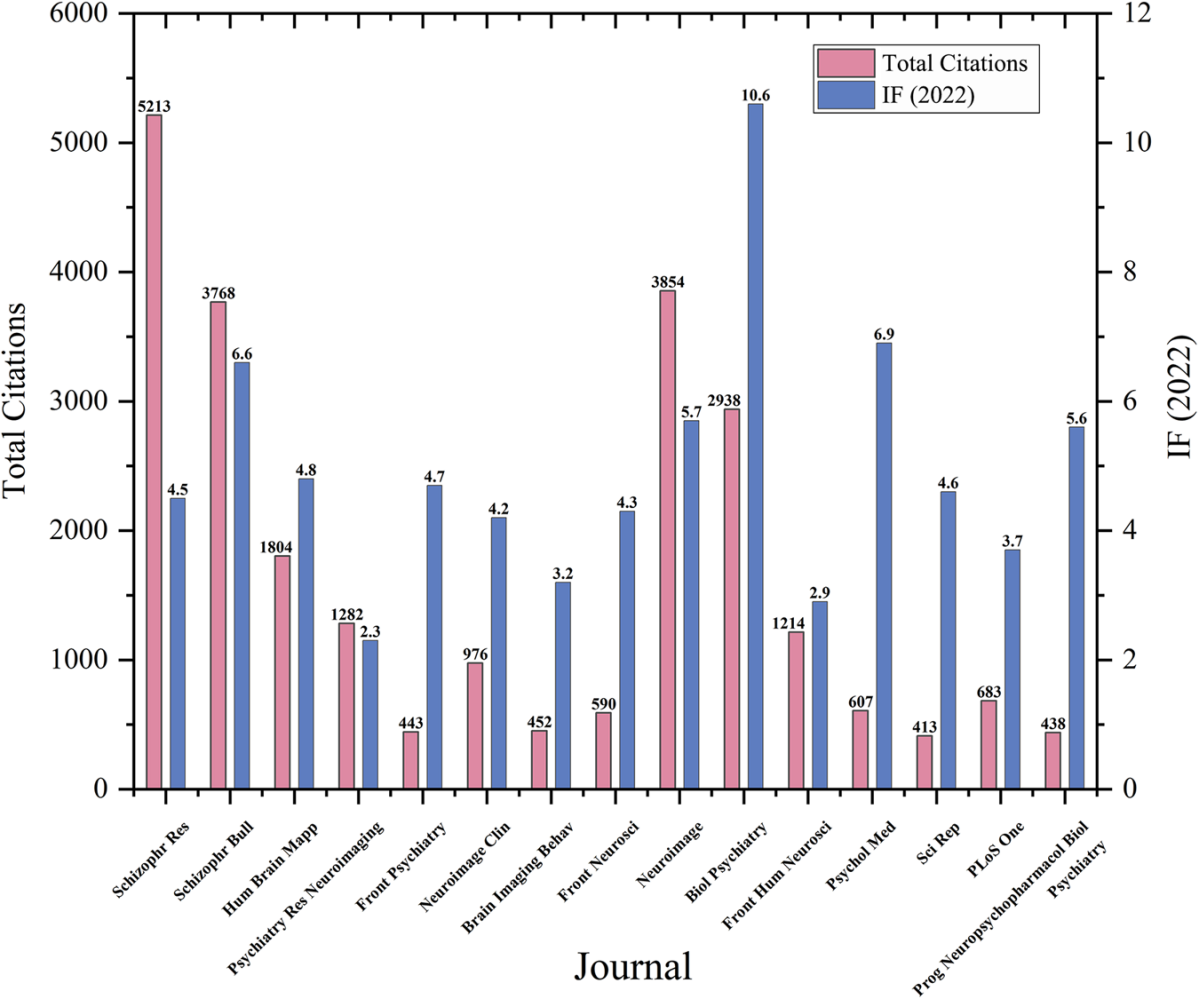
The top 15 productive journals for resting-state fMRI research in schizophrenia. Journals are listed according to the number of publications.

**Fig. 6:**
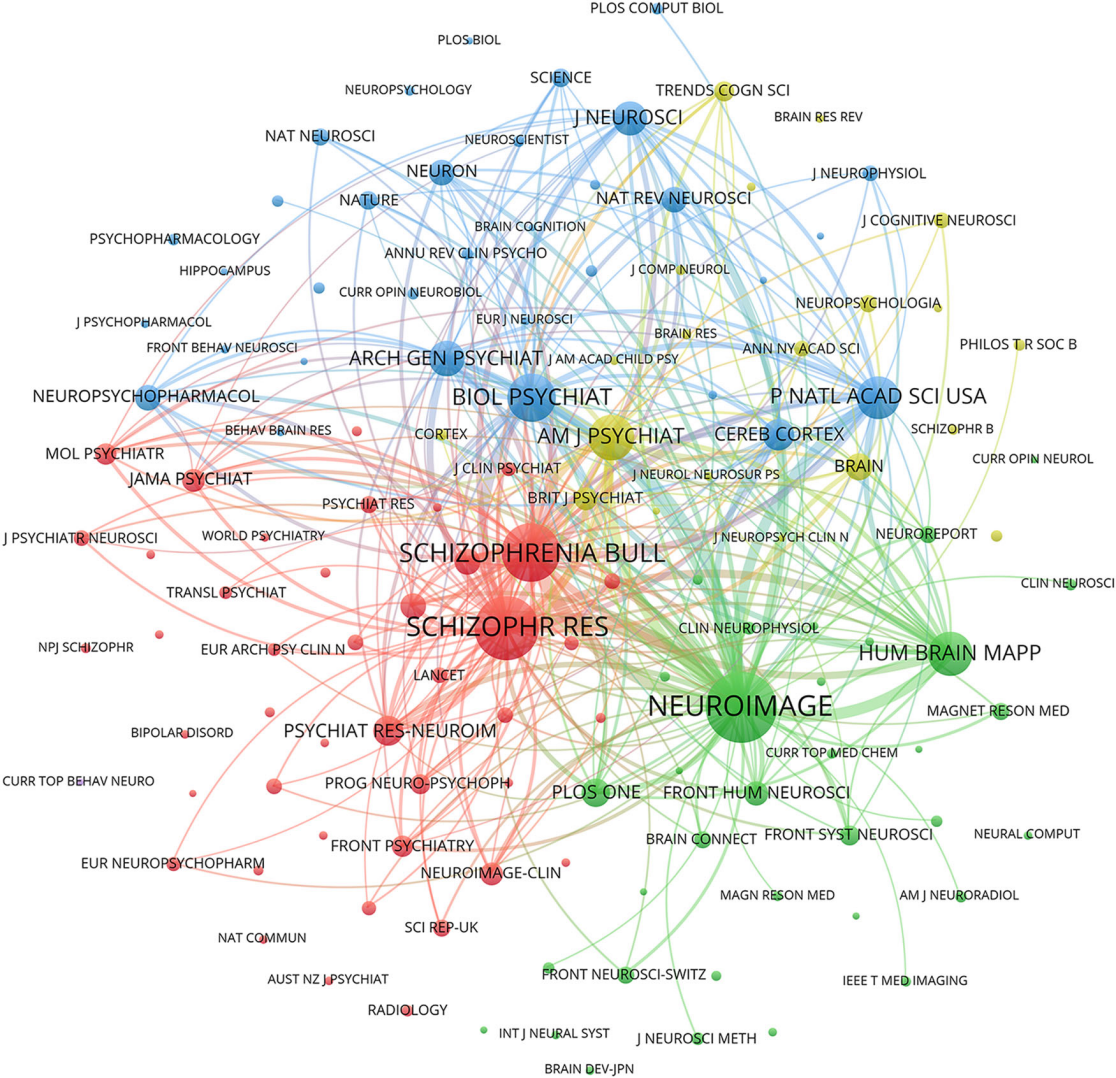
Journal co-citation map in the field of schizophrenia research based on resting-state fMRI. Each node represents one journal, with larger nodes indicating more citations. Lines between nodes demonstrate the number of their published documents cited by the same third document, with thicker line indicating a higher number of co-citations between them.

The 15 most productive journals for resting-state fMRI research in schizophrenia are listed in Table S5 and Fig. 5. Schizophrenia Research led with the largest number of relevant publications, totaling 105. Additionally, Schizophrenia Bulletin contained a total of 61 publications. Furthermore, Human Brain Mapping, Psychiatry Research-Neuroimaging, and Frontiers in Psychiatry also featured a significant number of publications, with counts of 47, 41, and 38, respectively. A co-citation network of journals in the field of schizophrenia research related to resting-state fMRI studies is shown in Fig. 6, and the network was constructed based on the citation relationships between journals. Additionally, the top 15 journals ranked by co-citations are presented in Table S6. Neuroimage had the highest number of co-citations (5138), followed by Schizophrenia Research (4125), Schizophrenia Bulletin (3378), Biological Psychiatry (2313), and American Journal of Psychiatry (2027). The majority of these journals belonged to JCR divisions categorized as Q1, highlighting their significant academic impact.

### Analysis of research hotspots and trends

**Fig. 7:**
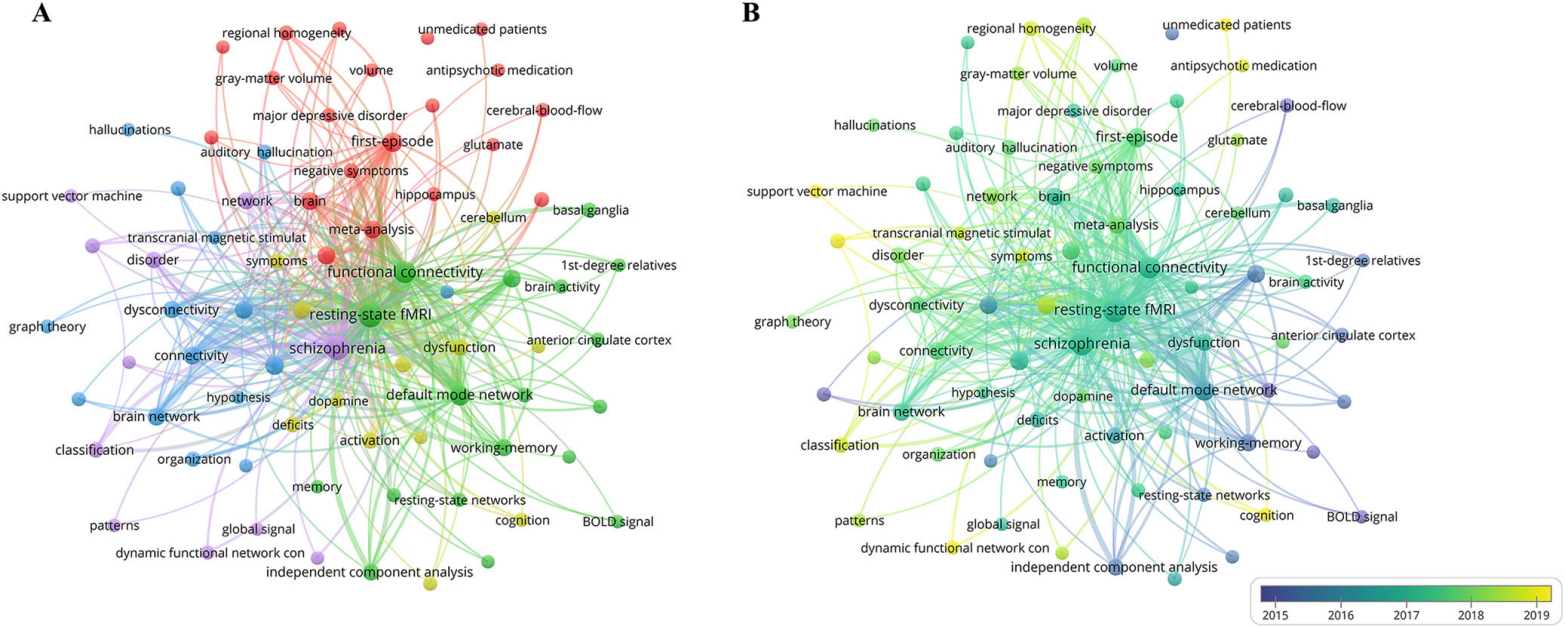
Analysis of research hotspots and trends in the field of schizophrenia research based on resting-state fMRI. **A** The keywords co-occurrence map. **B** Overlay visualization map of keywords’ co-occurrences.

A keyword co-occurrence analysis was conducted to investigate the research trends and prominent areas of study within the field. The resulting co-occurrence network was presented, focusing solely on keywords that occurred more than 20 times, leading to the classification of 76 keywords into five distinct clusters denoted by different colors (Fig. 7A).

Cluster 1 (in red, comprising 20 items) concentrated on the medication of schizophrenia and structural abnormalities, featuring keywords such as drug-naive schizophrenia, antipsychotic medication, and gray matter volume. Cluster 2 (in green, with 18 items) centered around functional brain networks and cognitive processes in schizophrenia, highlighting terms like default mode network, independent component analysis, and working memory. Cluster 3 (in blue, consisting of 15 items) emphasized neural connectivity, hallucinations, and interventions like transcranial magnetic stimulation, with primary keywords including connectivity, auditory hallucination, and transcranial magnetic stimulation. Cluster 4 (in yellow, including 12 items) focused on dysfunction, dopamine-related aspects, and cognitive performance in schizophrenia, featuring frequently used terms such as dysfunction, dopamine, and cognition. Finally, cluster 5 (in purple, encompassing 11 items) represented an interdisciplinary approach and the utilization of machine learning in schizophrenia research, with key terms including brain connectivity, machine learning, and dynamic functional network connectivity.

In Fig. 7B, an overlay visualization map assigned varying colors to keywords based on their average year of occurrence, with darker shades indicating early popular keywords like resting-state fMRI, schizophrenia, and BOLD signal, while lighter shades represented recently popular terms such as support vector machine, antipsychotic medication, and graph theory-related concepts.

## Discussion

In this study, the VOSviewer software was utilized to provide a comprehensive overview of the evolution of resting-state fMRI research in schizophrenia over the past 25 years. Leading countries/regions, institutions, authors, and journals contributing to research in resting-state fMRI in schizophrenia were identified. Additionally, cooperation networks among countries/regions, institutions, and authors, as well as journal co-citation networks, were analyzed. Keyword co-occurrence analysis was conducted to reveal coherent connections and highlight hotspots and trends in resting-state fMRI studies on schizophrenia.

The publication output concerning resting-state fMRI studies associated with schizophrenia exhibited a consistent upward trajectory from 1998 to 2022. This growth was primarily driven by several factors. Firstly, schizophrenia, being a complex syndrome, consistently remained a focal point of investigation^29–32^. Secondly, the non-invasive nature of resting-state fMRI rendered it a valuable tool for comprehensively assessing brain function, thus significantly advancing research in this area^33–35^. Thirdly, the urgent need to investigate the causes and treatments of schizophrenia led to a significant expansion in the existing body of literature^36–38^. Although there was a decline in publications in 2020, likely due to disruptions caused by the COVID-19 pandemic^39^, there was subsequent recovery in 2021 and 2022. This rebound can be attributed to the reinstatement of research funding and the adoption of new research methodologies and technologies, which continued to drive academic progress in this field.

China and the United States led in both total publications and citation counts in this field, with the institutions that made the largest contributions primarily located in these two countries. While China had slightly more publications than the United States, it only had half as many citations, which could be attributed to differences in the timing of when the two countries began research in this field. Additionally, international collaboration played a crucial role in resting-state fMRI studies related to schizophrenia, with collaborations between multiple countries/regions indicating that schizophrenia research became a global endeavor. China and the United States were also at the forefront of cooperation in this field. Active international collaboration facilitated the integration of global expertise and resources, promoting the continuous development of research. Therefore, greater collaboration networks among more nations/regions were established.

Among the top 15 productive institutions, 8 were located in China, and 5 were in the United States, indicating that China and the United States were the primary domains in the field. The high productivity of these institutions could be attributed to their strong research teams, abundant talent resources, effective research strategies, and positive academic culture. Regarding inter-institutional cooperation, scholars from all organizations tended to engage more in domestic collaboration than international collaboration. Furthermore, geographical patterns were evident, with inter-institutional collaboration primarily occurring among institutions in the United States. As the country with the largest schizophrenia population^40^, Chinese research institutes should actively engage in collaborations with other institutions.

Analyzing published articles and joint citations objectively evaluated the significant contributions and impacts of authors. Vince D. Calhoun from the United States stood out for his substantial research output and citation frequency, focusing on functional connectivity in schizophrenia using resting-state fMRI^41–43^. While China had a high paper output, Chinese scholars were less prominent among authors with the most co-citations, likely due to China’s relatively late entry into the field. Employing co-authorship and author co-citation networks helped visualize author influence, tracked field evolution, and identified collaboration opportunities, with network dynamics changing as new researchers emerged or field shifts occurred. Several journals were influential in the field of schizophrenia research, such as Neuroimage, Schizophrenia Research, Schizophrenia Bulletin, Biological Psychiatry, and the American Journal of Psychiatry. These journals promoted the development of schizophrenia research and the dissemination of knowledge. Future research could further explore the citation relationships between these journals to gain a more comprehensive understanding of the academic dynamics and development trends in this field.

Our bibliometric analysis identified five distinct clusters that explored research hotspots and potential trends in resting-state fMRI research related to schizophrenia, highlighting the multifaceted nature of the field. Cluster 1 focused on the medication and structural aspects of schizophrenia, suggesting that resting-state fMRI research commonly targeted specific patient subgroups associated with medication. In fact, the resting-state fMRI alterations observed in patients with different medication-related schizophrenia subgroups exhibited distinct characteristics^44,45^. Moreover, it is noteworthy that research in resting-state fMRI associated with schizophrenia often integrated multimodal approaches, simultaneously exploring brain structure alterations^46,47^. Cluster 2 centered on functional brain networks and cognitive processes. Studies investigating functional network disturbances in schizophrenia reported alterations in a wide variety of systems, including the default mode network, associated with introspective processing^48^, and fronto-parietal networks implicated in cognitive function^49^. Cluster 3 mainly included neural connectivity, hallucinations, and interventions like transcranial magnetic stimulation. Hallucinations are a core characteristic of schizophrenia, and transcranial magnetic stimulation may provide a viable treatment option for schizophrenia patients with hallucinations^50^. For cluster 4, the prominent keywords encompassed dysfunction, dopamine-related aspects, and cognitive performance, likely derived from the theory of dopamine function and its association with cognitive deficits in schizophrenia^51^. Cluster 5 represented the interdisciplinary approach and the application of machine learning in schizophrenia research, which played significant roles in the diagnosis, recovery, and quality of life outcomes for individuals with schizophrenia^52,53^.

The overlay visualization map highlighted the temporal evolution of research themes. Keywords such as support vector machine, antipsychotic medication, and transcranial magnetic stimulation gained recent attention due to advancements in machine learning, increased interest in the efficacy of antipsychotic medication, and the emergence of non-invasive brain stimulation techniques. These trends provided valuable insights for directing future research in diagnosing and treating schizophrenia using resting-state fMRI.

While resting-state fMRI provides valuable insights into brain connectivity and functional networks, its translation into clinical practice remains a challenge. Recent initiatives have introduced various translational methods involving resting-state fMRI, such as the adoption of new transdisciplinary diagnostic tools in schizophrenia. For example, the abnormal patterns uncovered by resting-state fMRI results, such as thalamic-auditory cortex-hippocampal connection defects or the abnormal cortical-striatal-cerebellar network connections identified in prior research, can serve as biomarkers for the preliminary diagnosis of schizophrenia^54,55^. Subsequent treatment with diagnostic medications, if successful in alleviating symptoms, can confirm the diagnosis of schizophrenia^56^. Moreover, the combination of clinical self-assessment scales with simultaneous fMRI acquisition explored the potential for translational cross-validation between clinical psychological tests and fMRI in psychiatry^57,58^, with a novel five-step approach proposed for translational validation based on prior explorative findings^59^. Furthermore, the potential for successful clinical application of this integration is significant, primarily due to its capability to guide pharmacological and neurostimulation treatments. These translation strategies hold the potential to synergize the strengths of traditional clinical methods with the complex neurobiological insights offered by resting-state fMRI, ensuring the reliability of diagnostics and treatment in schizophrenia.

Several limitations should be noted in this study. Firstly, the study exclusively relied on the Web of Science database as the primary data source. Although Web of Science is widely recognized for conducting literature bibliometric analyses across various disciplines, it may not have comprehensively represented all relevant literature within the studied field. Therefore, future research should encompass additional databases to obtain a more comprehensive depiction of research related to the use of resting-state fMRI in the domain of schizophrenia. Secondly, the study’s search was constrained to publications in the English language, potentially excluding important literature in other languages. Finally, while the detailed search strategy aimed to ensure the inclusion of a wide range of pertinent literature, certain relevant studies might have been omitted due to variations in terminologies and phrasing employed within the diverse landscape of this research field.

In conclusion, this bibliometric analysis summarized key trends in resting-state fMRI research on schizophrenia, spanning its historical development, current status, and future trends. China and the United States contributed significantly, with prominent institutions such as Central South University and the University of New Mexico. Research hotspots and trends were identified through keyword co-occurrence analysis, offering valuable insights for guiding future research in schizophrenia diagnosis and treatment using resting-state fMRI.

### Supplementary information

Supplementary Material

Dataset 1
