# Supplementary material for: Mapping the landscape: a bibliometric analysis of resting-state fMRI research on schizophrenia over the past 25 years
Source: Schizophrenia (Heidelb). 2024 Mar 15;10(1):35. doi: 10.1038/s41537-024-00456-2 (PMC10942978; doi:10.1038/s41537-024-00456-2)
Supplement: Supplementary file 1 — Supplementary Material [file 41537_2024_456_MOESM1_ESM.docx]

**Supplementary Material**

**Mapping the Landscape:** **A Bibliometric Analysis of Resting-State fMRI Research on Schizophrenia Over the Past 25 Years**

Linhan Fu^1, 2#^, Remilai Aximu^1, 2#^, Guoshu Zhao^1, 3#^, Yayuan Chen^1^, Zuhao Sun^1^, Hui Xue^1^, Shaoying Wang^1^, Nannan Zhang^1^, Zhihui Zhang^1^, Minghuan Lei^1^, Ying Zhai^1^, Jinglei Xu^1^, Jie Sun^1*^, Juanwei Ma^1*^, Feng Liu^1*^

**Affiliations:**

^1^Department of Radiology and Tianjin Key Laboratory of Functional Imaging, Tianjin Medical University General Hospital, Tianjin 300052, China

^2^School of Medical Imaging, Tianjin Medical University, Tianjin, 300070, China

^3^School of Medicine, Nankai University, Tianjin, 300071, China

^#^These authors contributed equally to this study

^*^**Corresponding authors:**

Feng Liu, PhD

E-mail: fengliu@tmu.edu.cn

Juanwei Ma, MD

E-mail: majuanwei@tmu.edu.cn

Jie Sun

jiesun19901212@163.com

Department of Radiology and Tianjin Key Laboratory of Functional Imaging, Tianjin Medical University General Hospital, No. 154, Anshan Road, Heping District, Tianjin 300052, China.

**Table S1.** Top 15 countries/regions with the most publications for resting-state fMRI research in schizophrenia.

| Index | Country/region | Count | Citation | Total link strength |
| --- | --- | --- | --- | --- |
| 1 | China | 399 | 12,235 | 244 |
| 2 | United States | 374 | 23,821 | 306 |
| 3 | England | 80 | 7,916 | 124 |
| 4 | Canada | 67 | 2,966 | 101 |
| 5 | Germany | 66 | 4,010 | 86 |
| 6 | Italy | 30 | 873 | 54 |
| 7 | Netherlands | 30 | 1,760 | 50 |
| 8 | Spain | 27 | 1,662 | 50 |
| 9 | Australia | 26 | 1,422 | 36 |
| 10 | South Korea | 25 | 788 | 18 |
| 11 | Japan | 23 | 369 | 14 |
| 12 | Taiwan | 20 | 993 | 25 |
| 13 | Switzerland | 20 | 828 | 23 |
| 14 | France | 19 | 651 | 29 |
| 15 | Norway | 14 | 833 | 12 |

**Table S2.** Top 15 institutions with the most publications for resting-state fMRI research in schizophrenia.

| Index | Institution | Count | Citation | Total link strength | Country |
| --- | --- | --- | --- | --- | --- |
| 1 | Central South University | 91 | 4,373 | 162 | China |
| 2 | University of New Mexico | 85 | 7,307 | 260 | United States |
| 3 | University of Electronic Science and Technology of China | 75 | 1,734 | 113 | China |
| 4 | The Mind Research Network | 72 | 4,468 | 234 | United States |
| 5 | Yale University | 70 | 7,710 | 178 | United States |
| 6 | Chinese Academy of Sciences | 61 | 4,299 | 155 | China |
| 7 | Sichuan University | 36 | 1,471 | 62 | China |
| 8 | Shanghai Jiao Tong University | 29 | 492 | 57 | China |
| 9 | Tianjin Medical University | 29 | 614 | 50 | China |
| 10 | Fourth Military Medical University | 27 | 731 | 35 | China |
| 11 | University of California, San Francisco | 26 | 1,033 | 127 | United States |
| 12 | University of Minnesota, Twin Cities | 26 | 1,471 | 102 | United States |
| 13 | Nanjing Medical University | 26 | 260 | 18 | China |
| 14 | King’s College London | 24 | 2,172 | 35 | England |
| 15 | University of Cambridge | 24 | 3,696 | 32 | England |

**Table S3.** Top 15 authors with the most publications for resting-state fMRI research in schizophrenia.

| Index | Author | Count | Citation | Total link strength | H-index |
| --- | --- | --- | --- | --- | --- |
| 1 | Vince D. Calhoun | 80 | 6,877 | 334 | 45 |
| 2 | Godfrey D. Pearlson | 44 | 5,972 | 204 | 25 |
| 3 | Jingping Zhao | 38 | 784 | 214 | 10 |
| 4 | Wenbin Guo | 35 | 936 | 199 | 20 |
| 5 | Cheng Luo | 29 | 648 | 174 | 17 |
| 6 | Feng Liu | 28 | 701 | 159 | 18 |
| 7 | Zhening Liu | 25 | 2,303 | 102 | 17 |
| 8 | Dezhong Yao | 25 | 617 | 151 | 15 |
| 9 | Daniel H. Mathalon | 22 | 900 | 179 | 17 |
| 10 | Hong Yin | 22 | 524 | 87 | 12 |
| 11 | Zhikun Zhang | 21 | 655 | 135 | 17 |
| 12 | Jijun Wang | 21 | 401 | 127 | 10 |
| 13 | Qiyong Gong | 20 | 797 | 100 | 14 |
| 14 | Huafu Chen | 20 | 358 | 123 | 14 |
| 15 | Su Lui | 19 | 999 | 107 | 12 |

*Note*: The H-index presented in this table was exclusively computed using publications on resting-state fMRI studies in schizophrenia authored by the individuals listed, as retrieved from Web of Science.

**Table S4.** Top 15 authors with the most co-citations for resting-state fMRI research in schizophrenia.

| Index | Author | Co-citation | Total link strength |
| --- | --- | --- | --- |
| 1 | Karl J. Friston | 497 | 11,246 |
| 2 | Vince D. Calhoun | 451 | 11,242 |
| 3 | Nancy C. Andreasen | 362 | 7,418 |
| 4 | Susan Whitfield-Gabrieli | 326 | 7,564 |
| 5 | Michael D. Fox | 321 | 8,314 |
| 6 | Wenbin Guo | 318 | 5,883 |
| 7 | Stanley R. Kay | 318 | 6,031 |
| 8 | Jonathon Power | 318 | 7,014 |
| 9 | Martijn van den Heuvel | 260 | 6,377 |
| 10 | Alexander Fornito | 253 | 6,882 |
| 11 | Neil Woodward | 245 | 5,482 |
| 12 | Alan Anticevic | 234 | 5,121 |
| 13 | Chao-Gan Yan | 234 | 4,336 |
| 14 | Stephen M. Smith | 220 | 5,256 |
| 15 | Su Lui | 215 | 4,791 |

**Table S5.** Top 15 productive journals for resting-state fMRI research in Schizophrenia.

| Index | Journal | Publication | IF (2022) | JCR  division |
| --- | --- | --- | --- | --- |
| 1 | Schizophrenia Research | 105 | 4.5 | Q2 |
| 2 | Schizophrenia Bulletin | 61 | 6.6 | Q1 |
| 3 | Human Brain Mapping | 47 | 4.8 | Q1 |
| 4 | Psychiatry Research-Neuroimaging | 41 | 2.3 | Q3 |
| 5 | Frontiers in Psychiatry | 38 | 4.7 | Q2 |
| 6 | Neuroimage Clinical | 34 | 4.2 | Q2 |
| 7 | Brain Imaging and Behavior | 26 | 3.2 | Q2 |
| 8 | Frontiers in Neuroscience | 26 | 4.3 | Q2 |
| 9 | Neuroimage | 26 | 5.7 | Q1 |
| 10 | Biological Psychiatry | 25 | 10.6 | Q1 |
| 11 | Frontiers in Human Neuroscience | 23 | 2.9 | Q3 |
| 12 | Psychological Medicine | 21 | 6.9 | Q1 |
| 13 | Scientific Reports | 18 | 4.6 | Q2 |
| 14 | Plos One | 16 | 3.7 | Q2 |
| 15 | Progress in Neuro-Psychopharmacology  Biological Psychiatry | 14 | 5.6 | Q1 |

Abbreviations: IF, impact factor; JCR, journal citation reports.

**Table S6.** Top 15 journals with the most co-citations for resting-state fMRI research in schizophrenia.

| Index | Journal | Co-citation | Total link strength | IF (2022) | JCR division |
| --- | --- | --- | --- | --- | --- |
| 1 | Neuroimage | 5,138 | 272,739 | 5.7 | Q1 |
| 2 | Schizophrenia Research | 4,125 | 236,945 | 4.5 | Q2 |
| 3 | Schizophrenia Bulletin | 3,378 | 195,273 | 6.6 | Q1 |
| 4 | Biological Psychiatry | 2,313 | 143,999 | 10.6 | Q1 |
| 5 | American Journal of Psychiatry | 2,027 | 118,253 | 17.7 | Q1 |
| 6 | Human Brain Mapping | 1,882 | 111,393 | 4.8 | Q1 |
| 7 | Proceedings of the National Academy of Sciences of the United States of America | 1,862 | 113,897 | 11.1 | Q1 |
| 8 | Archives of General Psychiatry | 1,260 | 75,987 | - | - |
| 9 | Journal of Neuroscience | 1,158 | 73,252 | 5.3 | Q1 |
| 10 | Cerebral Cortex | 966 | 61,053 | 3.7 | Q2 |
| 11 | Psychiatry Research-Neuroimaging | 881 | 56,465 | 2.3 | Q3 |
| 12 | PLOS ONE | 822 | 48,414 | 3.7 | Q3 |
| 13 | Brain | 673 | 39,697 | 14.5 | Q1 |
| 14 | Neuroscience & Biobehavioral Reviews | 665 | 42,351 | 8.2 | Q1 |
| 15 | Psychological Medicine | 650 | 40,020 | 6.9 | Q1 |

*Note*: the name of Archives of General Psychiatry was changed to JAMA Psychiatry in 2013.

Abbreviations: IF, impact factor; JCR, journal citation reports.
